# Reduction of the Hip-Bone by Manipulation; or, Fragments of the Literature of This Method

**Published:** 1857-11

**Authors:** Frank Hastings Hamilton


ART. II.—*Reduction of the Hip-Bone by Manipulation; or, Fragments of the Literature of this Method.* Compiled by FRANK HASTINGS HAMILTON, M. D.

"If the thigh-bone be luxated inward, and the patient young and of a tender constitution, it may be reduced by the hand of the chirurgeon, viz: he must lay one hand on the thigh, and the other on the patient's leg; and having somewhat extended it toward the sound leg, he must suddenly force the knee up toward the belly, and press back the head of the femur into its acetabulum, and it will knap in. For there is no need of so great extension in this kind of luxation; for the most considerable muscles being upon the stretch, the bowing of the knee as aforesaid reduceth it. Yet in rough bodies it may require stronger extension: and in that case the patient must be laid upon a table flat on his back; and a pin of a good thickness ought to be fixed in the middle of the table, to stand up between his legs close to that inguen, that the extension may be made thereby. During which the extension must be made by two men, the one pulling him by that arm-pit, the other by the leg; which latter extension must be made obliquely toward the sound leg."—*Eight Chirurgical Treatises. By Richard Wiseman, Serjeant-Chirurgeon to King Charles II. London:* 1676. *Book vii., Chap.* 8.

"If the bone be dislocated inward, and the patient young and tender, it may be reduced by hand; in order to which the operator must lay one hand upon the thigh, and the other upon the leg, and then suddenly forcing the knee up to the belly, and pressing the head of the femur back, the bone will slip into the acetabulum. In bodies where stronger extension is required, the patient is to be laid upon his back on a table, a thick pin being fixed in the middle, which rising up betwixt his legs is to be placed close to that inguen, where the protuberant bone is lodged; then one is to pull, taking hold of the armpit, and another is to extend by the leg obliquely toward the sound leg."—*A System of Rational and Practical Surgery. By Richard Bovlton, late of Brazen-Nose College, in Oxford. London:* 1713, *p.* 346.

"The manner of replacing the Dislocation, either inward or outward, is by laying the patient supine, or with his face upward upon a table, in which for the better extension or stay of the body, a wooden pin, about a foot and a-half long, is fixed, which is to bear close up the twist, or against the groin on the lame side; then fastening a strong towel or napkin about the knee, with another below, a third being beforehand put up to the groin, round about the hip, against which the pin aforesaid is to rest; after which, taking fast hold of the ends of the said towels, the extension, as also the counter-extension, is begun, by several strong arms, whilst one likewise, at each armpit, may farther the same, not only steadying of the body, but augmenting the counter stretch when it is wanting: During which, so soon as the surgeon perceives the bone moving out, let him take his opportunity, giving order to the extenders below, suddenly to lift up the patient's thigh toward his belly, pressing with his hands, either to the right or left, as the situation of the same requires, and therewith force back its head toward the acetabulum, whereunto it will, flipping over the tip of the cartilage, snap sometimes with a loud noise.

"But supposing the processus, or prominency locked under the glutei, or muscles of the buttock, the former posture is to be reversed, and the body laid prone, in like manner or on the face, the pin rising up, on the side of the twist, which, after suitable extension as before, the surgeon, lifting up the femur, or thigh-bone, or rather giving orders to the extenders so to do, with his hands presseth down the head into its seat aforesaid.

"The like where it is forced to the outside, the extension, elevation and pressure, being made to the contrary, that the bone may be more certainly and securely conducted to the cavity it was flown from; in which nothing but a right and due knowledge of the skeleton, in its several articulations, assisted also at the same time by a diligent comparison of the limbs, the the sound with the lame, can, as we have already more than once observed, render you complete or dexterous in this part of your profession.

"Where the extension by the hand proves insufficient, the tackle and pullies are wanted, to supply the deficiency thereof; notwithstanding which, though some very eminent practitioners have been concerned therein, I have more often known the dislocations of this kind still left unfinished, than those of the humerus, or shoulder-bone.

"An officer, at the Red-Lion in Red-Cross Street, a young sprightly fellow, being thrown from his horse, in the fall displaced this bone; finding himself unable to stand upon his leg, he was carried up to his chamber, and put into a bed; after which they sent for me, who, upon examination, plainly felt the head of the bone in the inguen, and the cup or cavity of the coxendix forsaken by the same.

"In order to its reduction, I called for the assistance of three strong fellows, at that time in the house; and, happening in his neighborhood, I sent also to Mr. Richard Bateman, then living just by, but he being from home, his servant came to me. I then asked for several strong towels, one of which

I drew up between his legs, close to the groin, but clear of the testis, each end of which was committed into the hands of two of the assistants before-mentioned, in order to a counter-extension; to two others, placed one above, the other below the joint of the knee, two more; and, as a farther help to the counter stretch, others were called in, who under each arm were to draw up the trunk, and keep the body from wriggling out of the way.

"Being thus set at their several pcsts, and all things provided I had occasion for, as the patient lay rather on the sound side, kneeling on the bed over him, I clapped my arms high up under the fleshy part of his thigh, ready for the work, and giving orders for the extension to be begun, both above and below, as I found the bone to move, with my arms, as aforesaid, I lifted it again to its acetabulum, where it flapped in very loudly, and perceptibly to us all.

"After this I applied a large defensitive plaister round the hip, with bolstering in the hollow of the twist, and rolled him securely up: The rest being nature's work, only directing him to lie still and quiet, and confining him to his bed for a month's time, which was not but with great difficulty complied with; for finding himsely so perfectly easy, eating, drinking and sleeping as well as before, having youth and an excellent well constituted temperament, he had no notion of the necessity of such confinement; and, unknown to me, had got up at the fortnight's end, of an evening, drinking and playing at cards with his companions. At about five week's end, coming into the yard, I saw him in the riding-place, laying his hands on a horse's back, leap upon the same; which he told me (and reasonably) was much easier to him, than mounting by the stirrups; in which, the heel making an angle with the hip, the head of the femur is brought out nearer to the brims of the cartilage, and in greater danger of slipping over the same, by stretching of the teres; Nor did he, as I could hear, ever make complaint of a weakness in the joint after.

"This case, with two others, in one of which, the head of the femur was beat backward, I do not blush to own, have been the only successful enterprizes of this nature, in which, I have myself, as principal, been concerned, which were all too performed singly by the strength of the arm, or without any other instrument."—*The Art of Surgery; by Daniel Turner, M. D. London:* 1742. *vol. ii, pp.* 339–42.

Heister recommends the use of hooks, or pulleys as a general rule; the method of using which he particularly describes; "or," he adds, "the reduction may be made by napkins, fastened around the extremity of the thigh like slings, much as in a luxation of the humerus; which will be more likely to succeed, if the knee be at the same time pressed inward by the hands."—*A General System of Surgery; by Dr. Laurence Heister, Prof'r of Physic and Surgery in the University of Helmstadt, &c, &c. London:* 1768. *Book III, Chap. x, p.* 186.

"*Two Cases of Dislocation of the Femur, with an account of the Method of Reduction.* By Mr. THOMAS ANDERSON, Surgeon in Leith.

I. "In Sept., 1772, Mr. Bruce, surgeon at Musselburgh, sent to me, desiring I would meet him at Lord Abercorn's coal-work, one of the colliers having dislocated his left thigh bone. I there found Messrs. Bruce and Stewart, surgeons in Musselburgh, and Messrs. Simpson and Clarkson, surgeons in Dalkeith, who were just begun to attempt the reduction by pullies. With these several trials were made; but the lacque round the knee slipping, it was taken off. By these means I had an opportunity of examining it. I found the left knee protruding three or four inches further than the right, and the one could not be brought within eight or ten inches of the other, the foot being turned out. When it was moved upward and downward, if done gently, he found little pain; but I observed, when it was nearly, or altogether extended, the head of the bone became fixed. And he complained more when it was in that situation, if any rotatory motion was attempted with the femur, which gave him no uneasiness when the thigh was brought up toward the abdomen. From the above appearance, it was certain the head of the bone was displaced from the acetabulum, and lodged downward and inward, in the large foramen of the ischium and pubis. I was convinced that attempting the reduction, in the common method, with the thigh extended, was improper, as the muscles were all put on the stretch, the action of which is perhaps sufficient to overbalance any extension we can apply. But by bringing the thigh, to near a right angle with the trunk, by which the muscles would be greatly relaxed, I imagined that the reduction might more readily take place, and with much less extension.

"When I made this examination, he was lying on a table on his back. I raised the thigh to about a right angle with the trunk, and, with my right hand at the ham, laid hold of the thigh, and made what extension I could. From this trial I found I could dislodge the head of the bone. At the same time that I did this, with my left hand at the head and inside of the thigh, I pressed it towards the acetabulum, while my right gave the femur a little circular turn, so as to bring the rotula inward to its natural situation; and, on the second attempt, it went in with a snap observable to the gentlemen standing around, but more so to the poor man, who instantly cried out he was well and free from pain. His knees could then be brought together; the legs were of the same length, and the foot in its natural situation. The knees were kept together for some time, with a roller, to confine the motion of the thigh; and in three weeks, he was at his work, without the least stiffness in the joint.

II. "A boy, eight years old, of a strong healthy constitution, while he happened to be carried on his sister's back, lost the hold he had of her neck, and fell to the left side. She, however, held him by the legs, which were round her waist, so as to occasion considerable stress to the parts. He was carried home and complained of the left thigh and haunch, which he said was from a fall from his sister's back. The parents being poor, and imagining it to be only bruised, were eighteen days before they called any assistance. At this time I found him lying in bed on his back, the fore part of the femur turned quite in, the knee lying on the right thigh, was fully four inches shorter, the leg turned out and considerable tension and swelling on the hip. From the appearance, I suspected a fracture at the neck of the bone; but on examining it was soon convinced of the dislocation, and that the head of the bone was lodged upward and backward from the acetabulum, in the concave part of the ilium where it joins the ischium. The smallest attempt to carry the thigh outward from the position in which it lay, gave him the most exquisite pain, and he could only allow it to be gently moved upward, if at the same time, the knee was kept over to the right side. From the motion made to discover the situation, he complained so much, that the reduction was not attempted at that time. The hip was fomented, rubbed with camphorated oil, and a poultice applied for that night. Next forenoon I called on him with two young gentlemen; he was placed across the bed, the thigh raised so as to form an acute angle with the trunk. In this situation the knee lay considerably over to the right side, and the leg was turned much outward. He was kept down by an assistant, while I laid hold, with both hands, above the knee, at the same time standing upon the side of the bed and pulling upward, I found I could move the head of the bone from the place where it was lodged; and upon making considerable extension, with my left hand, which I brought inward. By this the femur made a circular turn, which directed its head toward the acetabulum, into which it went with a sensible noise. The boy immediately cried out, that he was well, and could allow the thigh to be moved gently in any direction.

The thighs were kept together for two weeks with a bandage, and, in three weeks, he could walk; but he complained of stiffness in the joint for a week or two afterward."—*Medical Commentaries, published at Edinburgh*, 1776, *vol. ii, pp.* 261–4.

(To be Continued.)

